# Enhanced BDNF and ROS in Mucosa of Lower Motor Neuron Lesioned Dog Bladder Following Somatic Motor Nerve Transfer

**DOI:** 10.3390/cells14060406

**Published:** 2025-03-11

**Authors:** Nagat Frara, Kais Jawawdeh, Dania Giaddui, Istvan P. Tamas, Ryan P. Gares, Elizabeth R. McGonagle, Brendan A. Hilliard, Mikhail A. Kolpakov, Lewis Bright-Rowe, Alan S. Braverman, Justin M. Brown, Michael R. Ruggieri, Mary F. Barbe

**Affiliations:** 1 Aging + Cardiovascular Discovery Center, Lewis Katz School of Medicine, Temple University, Philadelphia, PA 19140, USA; istvan.tamas@temple.edu (I.P.T.); elizabeth.mcgonagle@temple.edu (E.R.M.); brendan.hilliard@temple.edu (B.A.H.); mikhail.kolpakov@temple.edu (M.A.K.); brightrowe@temple.edu (L.B.-R.); alan.braverman@temple.edu (A.S.B.); 2 Center for Translational Research, Lewis Katz School of Medicine, Temple University, Philadelphia, PA 19040, USA; kais.jawawdeh@temple.edu (K.J.); dgiaddui96@gmail.com (D.G.); ryan.gares@temple.edu (R.P.G.); michael.ruggieri@temple.edu (M.R.R.S.); 3 Department of Neurosurgery, Massachusetts General Hospital, Boston, MA 02114, USA; jmbrown@mgh.harvard.edu

**Keywords:** neurotrophic factor, bladder mucosa, detrusor muscle, peripheral nerve injury, plasticity, reactive oxygen species, inflammation, axonal density

## Abstract

Neurotrophic factors and reactive oxygen species (ROS) modulate neuronal plasticity. In a model of a lower motor neuron lesioned bladder, somatic nerve transfer was used as a reinnervation strategy. Levels of neurotrophins, ROS, and TNF-α in bladder mucosa and muscle layers collected from three groups of adult female dogs: (1) Decentralized, via bilateral transection of coccygeal and sacral spinal roots, lumbar 7 dorsal roots, and hypogastric nerves, then 6–21 mo recovery; (2) reinnervated (ObNT-Reinn), after similar decentralization for 12 mo, then bilateral obturator-to-vesical nerve transfer and 8–12 mo recovery; and (3) Controls. In mucosa, BDNF and ROS levels were highest in ObNT-Reinn bladders, GDNF and TNF-α levels were restored to Control levels in ObNT-Reinn bladders (lowest in Decentralized). NT-3 and ARTN were lower in ObNT-Reinn and Decentralized bladders versus Controls. In muscle, ROS was lower in ObNT-Reinn muscle versus Controls. BDNF mucosa levels correlated with bladder axonal density and detrusor layer thickness; and GDNF mucosal correlated with bladder contraction after vesical or transferred obturator nerve electrical stimulation, as did BDNF and GDNF muscle levels. The increased BDNF and GDNF in bladders that underwent somatic nerve transfer with subsequent recovery suggest that BDNF and GDNF may help promote the reestablishment of bladder innervation.

## 1. Introduction

Spinal injuries have been linked to a number of bladder pathologies [1], and consequently, poor quality of life [2]. There is an urgent need for effective treatments [2,3,4]. Peripheral nerve transfer is a common strategy to restore innervation when motor control of a target organ is lost due to injuries to nerves, spinal roots, or the spinal cord [5,6,7]. The survival of sensory and motoneurons requires new synaptic contacts with peripheral targets [8,9,10,11]. Although still under investigation, nerve transfer strategies to restore bladder reinnervation and emptying function are anticipated to improve the quality of life of patients with such injuries [2,12,13,14,15,16].

In preclinical investigations utilizing a canine model of a lower motor neuron lesioned urinary bladder, our laboratory has developed surgical approaches to reinnervate the bladder to regain function [4,17,18,19]. Our most recent findings showed that animals decentralized for one year began to show urination postures at 3–4 months that were maintained at 12 months after nerve transfer surgeries in which a subdivision of the obturator nerve was transferred to bladder vesical branches [19]. Increased detrusor pressures upon the stimulation of spinal root segments matched the origin of the transferred nerve in 37% of animals. However, 63% of the reinnervated animals showed less robust contractions. Similar less robust contractions after reinnervation have been reported in other studies [12,13,14,15,16,20,21]. An incomplete recovery could be due to the loss of spinal cord motoneurons, loss of bladder intramural ganglion neurons, or limited regrowth of motor fibers [8,22]. Reinnervation of the bladder is certainly possible based on results from several prior studies [23]. Support for this potential includes: (1) adult lumbar motoneurons remain viable up to one year after nerve injury [24,25,26,27]; (2) axonal regrowth to the bladder can occur through repaired nerves and roots [4,17,18,28,29,30]; and (3) sprouting occurs at distal ends of transected nerves innervating the bladder [17,28,31,32,33], similar to nerve sprouting in skeletal muscle after somatic nerve injury [34].

Among promising therapeutic means to improve nerve regeneration is the provision of neurotrophic factors [35]. Neurotrophic factors implicated in neuronal growth, survival and maintenance include: (1) neurotrophins, e.g., brain-derived neurotrophic factor (BDNF), nerve growth factor (NGF), and neurotrophin-3 (NT-3); (2) glial cell line-derived neurotrophic factor family, e.g., glial cell line-derived neurotrophic factor (GDNF) and Artemin (ARTN); and (3) neuropoietic/neuro-differentiation cytokines, e.g., ciliary neurotrophic factor (CNTF) and tumor necrosis factor alpha (TNF-a) [36]. Neurotrophins play roles as messengers between peripheral effector tissue and nerves [37], and promote the survival of injured motoneurons [38,39]. Previously, in our canine model, we found that delivering BDNF to the site of coaptation induced significant axonal sprouting and outgrowth into connective tissues surrounding a root repair site [17]. In a rat model of cavernous nerve electrocautery-induced erectile dysfunction, the injection of stem cells oversecreting BDNF into the corpus cavernosum promoted cavernous nerve regeneration, inhibited fibrosis, and enhanced erectile function [40]. The application of BDNF and GDNF at a nerve lesion site promotes spinal motoneuronal survival and axonal regeneration [41,42,43,44], and continued administration of GDNF enhances axonal regrowth [11]. Neurotrophic factors also modulate neuronal plasticity in association with reactive oxygen species (ROS) activity [45,46,47,48,49].

In future studies, we seek to improve functional innervation of the bladder using neurotrophic factors. In this exploratory study, we sought to identify potential candidates by examining bladder tissues for changes in mRNA or protein expression of several neurotrophic factors after decentralization and/or somatic nerve reinnervation procedures, compared to intact bladders. We examined: (1) select neurotrophins, BDNF, NGF and NT-3; (2) the GDNF family members, ARTN and GDNF; (3) neuropoietic/neuro-differentiation cytokines, CNTF and TNF-α; (4) and nicotinamide adenine dinucleotide phosphate (NADPH)-dependent ROS production.

## 2. Materials and Methods

### 2.1. Animals

**Figure 1:**
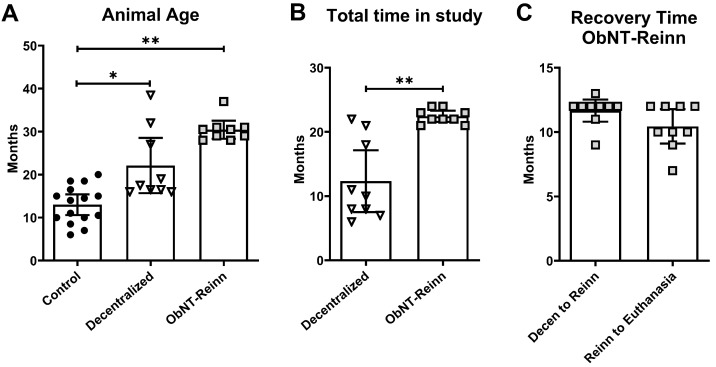
Animal age, total time in study, and post-operative recovery time. (**A**) Ages (in months) of all animals in the three dog groups, Control (*n* = 15), Decentralized (*n* = 9), and ObNT-Reinn (*n* = 9), where *n* = number of animals. (**B**) Total time in study (in months) of the Decentralized and ObNT-Reinn animals at onset of experiment when animal entered study until tissue collection and euthanasia (**C**) Postoperative recovery time for ObNT-Reinn animals provided as post-decentralization recovery time before reinnervation procedures (Decen to Reinn) and post-reinnervation and before bladder tissue collection and euthanasia (Reinn to Euthanasia). Shown are means ± 95% CI. *: *p* < 0.05 and **: *p* < 0.01, compared between groups as shown. A Kruskal–Wallis ANOVA followed by Dunn’s multiple comparisons post hoc test was used to compare the age between the three groups shown in panel (**A**); a parametric unpaired t test with Welch’s correction was used for data shown in panel (**B**); and a nonparametric Mann–Whitney two-tailed test was used for data shown in panel (**C**).

Prior to the onset, these studies were approved by the Institutional Animal Care and Use Committee (protocol number was 5043) according to the guidelines of the National Institute of Health for the Care and Use of Laboratory Animals, the United States Department of Agriculture, and the Association for Assessment and Accreditation of Laboratory Animal Care. This study used a total of 33 mixed-breed hound female dogs, including 15 Controls (8 sham-operated, 5 unoperated, and 2 sham-operated derived from other larger studies focusing on heart failure), 9 Decentralized, and 9 reinnervated (ObNT-Reinn). Allocation of the animals within each experimental group was performed randomly. The animals were 6–8 months of age at the onset of the experiment, when they entered the study, weighing 20–25 kg (Marshall BioResources, North Rose, NY, USA), and were group housed according to the institution’s standard husbandry with 12 h exposure to light/dark cycles. The age of each animal in months in all dog groups, calculated from the period between date of birth to euthanasia, is shown in Figure 1A.

### 2.2. Bladder Decentralization Surgery

Eighteen dogs underwent decentralization surgeries performed as previously described and illustrated [50,51]. An anatomical diagram of the decentralization surgical process, versus intact state, is shown in Supplemental Figure S1A,B. Briefly, after anesthesia, all 18 animals were subjected to laminectomy that extended from sixth lumbar (L) vertebrae to the second sacral(S) vertebrae in order to expose the lower spinal cord and identify the sacral spinal roots. We achieved decentralization of the bladder via bilateral transection of all posterior and anterior roots caudal to L7, and the posterior roots of L7. In 11 of the animals, the spinal ganglia were completely removed, as previously described, to reduce sensory driven squat and void behaviors (inflammation driven) [19,50], while in the other 7 (our initial animals), these ganglia remained intact. Hypogastric nerves were accessed in the abdomen and bilaterally transected in all decentralized animals (both Decentralized and ObNT-Reinn groups). For complete separation, 10 to 15 mm sections were removed from each transected root or nerve. Animals in the Decentralized only group (*n* = 9) were provided with a postoperative recovery period of 6–22 months before bladder tissue collection and euthanasia (Figure 1B). While animals in the ObNT-Reinn group (*n* = 9), were provided 9–13 months as post-decentralization recovery time before reinnervation procedures, and then 7–12 months recovery time (Figure 1C). The duration of recovery time was based on the return of voiding behaviors or the lack thereof [19].

### 2.3. Bladder Reinnervation Surgery Using Nerve Transfer

Nine of the previously decentralized animals were deeply anesthetized before abdominally accessing and identifying the donor obturator nerves. Only a small portion of the obturator nerve, approximately one-third, was initially split and longitudinally transected, before being transferred and sutured end-to-end to the transected vesical branch of the inferior hypogastric plexus, bilaterally, as previously described and illustrated [19,51]. An anatomical diagram of the reinnervation surgical process is shown in Supplemental Figure S1. The remaining two-thirds of the obturator nerve were left intact to preserve the function of muscles of the hind limb. A nerve connector (Axoguard, Axogen Corp, Alachua, FL, USA) and sealant (Tisseel fibrin, Baxter, Deerfield, IL, USA) were used to maintain and reinforce the transferred nerve’s coaptation site. The ObNT-Reinn animals were given a recovery time of 7–12 months after reinnervation and before euthanasia and tissue collection. The ObNT-Reinn animals also underwent reinnervation of the anal sphincter via a pudendal nerve transfer, results of which have been reported [19]; however, here, we focused only on the bladder outcomes.

### 2.4. Postoperative Care

Subcutaneous administration of buprenorphine (0.03–0.05 mg/kg) was provided twice a day for 2 days postoperatively after each surgical procedure. Because of the sacral root transection and loss of function in both vesical nerves, the Decentralized and ObNT-Reinn animals had urinary incontinence. Therefore, the Crede’ maneuver was performed twice daily on Decentralized animals for bladder emptying. The frequency of squat and-void postures was recorded for 24 h at monthly intervals pre- and post-operatively, as previously reported [19]. Urinalysis results have been previously reported [50]. Although, all Decentralized and ObNT-Reinn animals had multiple instances of culture-confirmed bacteriuria, no catheterized urine specimens from animals collected before any surgery were culture positive [50].

### 2.5. Bladder Tissue Collection for Biochemical Assays

At study completion, whole bladders were harvested immediately before the terminal euthanasia and while animals were still deeply anesthetized, as described [50]. Bladders were removed with distal ends of the ureters still attached for orientation purposes. Then, animals were immediately euthanized using a terminal dose of pentobarbital sodium, 86 mg/kg, with phenytoin sodium, 11 mg/kg, i.v. Dissections of bladder specimens for biochemical assays were performed in a cold room (0–5 °C) with the tissues kept on ice during the dissection process. The bladder mucosa was dissected free from the underlying muscle layers (mucosa-denuded) using sharp micro scissors and 5x magnifying loops. All the mucosa and muscle tissues used in this study were dissected from the middle part of the bladder, at least 1 cm above the ureteral orifices.

### 2.6. Neurotrophic Factor’ mRNA Gene Expression

Portions of bladder tissue from three Decentralized and three Control animal bladders were preserved in RNALater (4,427,575, ThermoFisher Scientific, Wilmington, DE, USA) and used for a pilot gene expression study to potentially inform future neurotrophic factor choices. Mucosal and detrusor muscle were separated by dissection using sharp micro scissors, forceps and 5× magnifying loops. The tissue was then flash frozen by adding it to a mortar cooled on dry ice then liquid nitrogen was added, and the tissue was ground to a fine powder using a dry ice-cooled pestle. The powdered tissue was transferred to a 1.5 mL microfuge tube cooled on dry ice and 1 mL of Trizol (15596018, Life Technologies Corporation, Carlsbad, CA, USA) was added to the powder and vortexed immediately. The tissue was vortexed twice more with a pause for 5 min between the vortexing intervals. Total RNA was prepared according to the Trizol manufacturer’s instructions: chloroform (200 µL) was added to each tube and the tubes vortexed three times for 5 s with a short pause in between. The sample was centrifuged at 16,000× *g* relative centrifugal force for 15 min at 4 °C. The upper aqueous phase was removed to a fresh microcentrifuge tube, 10 µg of RNase free glycogen (AM9510, Invitrogen, ThermoFisher Scientific, Carlsbad, CA, USA) was added to the mucosa samples and 5 µg was added to the muscle samples and RNA was precipitated by adding 500 µL of isopropanol, vortexing briefly and allowed to stand on ice for 30 min. The precipitated RNA was pelleted at 16,000× *g* at 4 °C for 15 min, the pellet was washed with RNase-free 75% ethanol, centrifuged again, air dried, and resuspended in 30 µL or 60 µL of RNase-free water. The amount of RNA was quantified by measuring the absorbance at 260 nm using the Nanodrop 2000 spectrophotometer (ThermoFisher Scientific, Carlsbad, CA, USA) as described in the Nanodrop 2 manufacturer’s manual. Sample assays were run in duplicate.

Any potential contamination of the RNA with DNA was reduced by treating the samples with DNase. A 20 µg sample of the RNA was digested with 2 U RNase-free DNase I (M0303S, New England Biolabs, Ipswich, MA, USA) in a volume of 100 µL according to the manufacturer’s instructions. The DNase was removed by adding 100 µL of phenol: chloroform: isoamyl alcohol, vortexing, centrifuging (12,000× *g*, 15 min, 4 °C), and pipetting off the aqueous phase which was placed in a fresh microfuge tube. The RNA was precipitated after adding glycogen 10 µL (50 µg) (AM9510, Invitrogen, ThermoFisher Scientific) by adding 10 µL of RNase-free Sodium Acetate, 280 µL of Ethanol and centrifuging at 12,000× *g* at 4 °C, the pellet was washed with RNase-free 75% ethanol, air dried, and resuspended in either 30 µL of RNase-free water for samples with higher concentrations of RNA or with 10 µL for samples with low RNA concentration. The RNA was quantified again using the Nanodrop 2000.

cDNA was transcribed from 610.5 ng of muscle RNA or 707.3 ng of RNA from the mucosa with random hexamer primers using superscript IV reverse transcriptase First-Strand Synthesis System (Invitrogen, ThermoFisher Scientific) according to the manufacturer’s instructions.

For the real time polymerase chain reaction, gene-specific Taqman qPCR for each gene were obtained from Applied Biosystems (ThermoFisher Scientific, Carlsbad, CA, USA) as detailed in Table 1. Real time PCR was performed in an Applied Biosystems 7500 instrument, using a two-temperature cycle: 95 °C for 15 sec and an annealing/extension temperature of 60 °C, for 40 cycles. We used the “absolute” values because there was large variation in the quantities of the b-actin reference gene used, suggesting that the levels of its expression were affected by the surgical procedure. This choice allowed us to readily compare the levels of expression between each of the growth factors examined.

### 2.7. Enzyme-Linked Immunosorbent Assays (ELISA)

Unfixed and flash-frozen mucosa and muscle samples were collected as indicated above, homogenized in either sterile, ice-cold, phosphate-buffered saline (PBS), or PBS containing fresh proteinase inhibitors (Complete EDTA free Protease Inhibitor tablets, 5056489001, Sigma-Aldrich, Inc., St. Louis, MO, USA). Homogenates were centrifuged at 12,000 rpm for 15 min at 4 °C. Supernatants were aliquoted and stored at −80 °C until they were assayed via ELISA, in duplicate, where they were thawed on ice. ELISA kits, used in accordance with manufacturers’ instructions were: Quantikine^®^ Total BDNF (DBNT00, R&D Systems Inc., Minneapolis, MN, USA), canine nerve growth factor (NGF, MBS738670, MyBioSource, San Diego, CA, USA), canine neurotrophin-3 (NT-3, MBS741186, MyBioSource), canine glial cell line-derived neurotrophic factor (GDNF, MBS744164, MyBioSource), canine Artemin (ARTN, MBS735757, MyBioSource), canine ciliary neurotrophic factor (CNTF, MBS742904, MyBioSource), and canine TNF-α (ECTNF, Invitrogen, ThermoFisher Scientific, Carlsbad, CA, USA). The sensitivity of the ELISA kits were as follows: BDNF was 1.35 pg/mL, NGF was 1.0 pg/mL, NT-3 was 1.0 pg/mL, GDNF was 1.0 pg/mL, ARTN was 1.0 ng/mL, CNTF was 1 pg/mL, and TNF-α was 2 pg/mL. Data (pg or ng of protein) were normalized to pg or ng per micrograms of total protein, determined using Pierce™ Bicinchoninic Acid Protein Assay Kit (23227, Pierce, ThermoFisher Scientific, Rockford, IL, USA).

### 2.8. Measurement of ROS Superoxide Production

ROS superoxide levels were measured in homogenized dog bladder mucosa and muscle tissues using previously described methods [52]. Briefly, flash-frozen mucosa and muscle samples were homogenized in buffer, and protein concentrations were measured. The chemiluminogenic substrate lucigenin (10,10′-dimethyl-9,9′-biacridinium, dinitrate, 14872, Cayman Chemicals, Ann Arbor, MI, USA) was utilized (5 µM) [52]. Total homogenates of each sample (25 µL) were added to each well of a 96-well microplate, in triplicate. Basal levels of ROS were determined by measuring the light emitted per well across 15 min using a luminometer plate reader maintained at 37 °C (GloMax^®^ Discover Dual Injectors with Pumps, GM3030, Promega, Madison, WI, USA). NADPH (fresh; sodium salt, 9000743, Cayman Chemicals) was injected into each well (final concentration of 100 µM) and the microplate was re-read over a 15 min period. Then, the superoxide scavenger, Tiron (4,5-dihydroxy-1,3-benzene-disulfonic acid, ab146234, Abcam, Waltham, MA, USA) was injected into each well (final concentration of 20 mM) and the microplate re-read over a 15 min period. The amount of superoxide produced was calculated as previously described [52] and the relative luminescence units (RLU) emitted over time is reported.

### 2.9. Immunohistochemistry on Full Thickness Bladder Specimens

After the terminal surgeries, in addition to the collection of specimens for biochemical assays, full thickness bladder specimens (approximately 3 cm^2^ in size) were also collected for histological studies. These tissues were fixed and then frozen sectioned as previously described [22]. To visualize the location of BDNF and TRK B/NTRK2 in the bladder tissues, immunohistochemistry was performed on subsets of sections (on slides) after an antigen retrieval step of 0.5% pepsin in 0.01N HCL for 15 min, blocked using 5% bovine serum albumin (BSA) for 30 min, and incubated with either an antibody against BDNF (GTX10832, GeneTex, Irvine, CA, USA; mouse monoclonal, 1:50 dilution) or TRK B (BDNF/NT-3 growth factor receptor, MBS822075, MyBioSource, rabbit polyclonal, 1:200 dilution), with the antibodies diluted with 2% BSA, overnight at room temperature. Slides were washed on a shaker with PBS and incubated with an Alexa Fluor 647 Goat anti-rabbit IgG (111-605-144, Jackson ImmunoResearch, West Grove, PA, USA), Alexa Fluor 647 Goat anti-mouse IgG (115-605-166, Jackson ImmunoResearch), or Alexa Fluor 488 Goat anti-rabbit IgG (111-545-144, Jackson ImmunoResearch), and diluted 1:100 with PBS for 2 h at room temperature. Sections of tissues were counterstained with DAPI and then coverslipped with 80% glycerol in PBS. Sections were imaged using a Nikon Eclipse E800 microscope (Nikon, Melville, NY, USA) equipped with a Jenoptik Graphax digital camera (Jenoptik, Jena, Germany) and imaging software (Bioquant Osteo, version 2024, Bioquant Image Analysis Corp, Nashville, TN, USA). The specificity of the antibody was assayed by incubating only with the secondary antibody.

### 2.10. Dihydroethidium (DHE) Detection of ROS in Tissue Sections

A subset of full thickness bladder specimens was snap frozen in O.C.T. (Compound Embedding Medium for frozen tissues, 23730571, Fisher Healthcare Tissue-Plus, Houston, TX, USA) in liquid nitrogen. Sections were frozen sectioned on a cryostat into 18-micron thick sections, placed onto poly-L-lysine-coated slides (P0425-72EA, Sigma-Aldrich, Inc., St. Louis, MO, USA), and stored in a −80 °C freezer until used. On the day of staining, slides were removed from the freezer and dried under a fan for 15 min. An equilibration buffer (50 µM MgCl_2_ in Hanks balance salt solution) was made fresh and warmed in a 37 °C water bath. DHE (12013, Cayman Chemical, Ann Arbor, MI, USA) was dissolved in a 5 mM stock solution of DMSO (light sensitive, so kept wrapped in foil). A DHE stain solution (2 µM) was prepared by adding 1 µL of the DHE stock solution to 2 mL of the warm buffer. Slides were placed into a damp incubation chamber (covered with foil) and approximately 100 µL of the buffer (without DHE) pipetted onto the top of each tissue section. Sections were incubated in the chamber for 30 min in a 37 °C oven. After the buffer incubation step, parallel sections were treated with 100 mM Tiron (ab146234, Abcam, Waltham, MA, USA) for 10 min, to serve as negative control stained sections. All slides were transported to a confocal microscope in the foil covered incubation chamber. Then, the DHE stain was added to each tissue section (approximately 100 µL per section), the slides were immediately coverslipped and imaged using a confocal microscope set at 520 nm excitation and 605 nm emission.

### 2.11. Statistical Analyses

Statistical analyses were performed using Prism version 10.2.3 (GraphPad Software, La Jolla, CA, USA). Data are presented as means with 95% confidence intervals (CI). For all data, the mean of each animal’s replicates were used in the statistical analyses. Samples from each dog bladder tissues were tested in duplicates for both gene and protein expression. The mRNA and ELISA data were analyzed using repeated-measures, mixed-effects, REML (Restricted Maximum Likelihood) model using the factors: *tissue type* and *surgical group*. This was followed by Tukey’s multiple comparisons for ELISA data, or Uncorrected Fisher’s LSD post hoc tests for mRNA gene expression data and ROS data, to determine differences between groups. *p* values were adjusted for multiple comparisons when applicable, and values of 0.05 or less were considered statistically significant for all analyses. Data were tested for normality using Shapiro–Wilk and Kolmogorov–Smirnov tests that were performed before any further tests and residuals were inspected. Data that had a normal distribution were analyzed using a parametric t test with Welch’s correction to compare two groups. If data did not pass the normality tests, a non-parametric Mann–Whitney test was used to compare between two groups, or Kruskal–Wallis ANOVA followed by Dunn’s multiple comparison post hoc tests were used to compare between three groups. Spearman’s rank correlation tests were used for relationships between biomarker data reported in this manuscript and previously reported histological changes in the bladder wall of these same animals [22], and previously reported functional electrophysiological data (specifically, maximum detrusor pressure) after stimulation of bladder nerves or nerve roots [19]; these data are provided in Supplemental Figure S3A–G. Results are reported as “r” in Table 2, Table 3, Table 4 and Table 5, with values of 0.4 to 0.59 (−0.4 to −0.59) considered as moderately positive (or negative) correlations, and values between 0.6 and 0.79 (−0.6 to −0.79) as strongly positive (or negative) correlations. This study is exploratory and did not test a prespecified statistical null hypothesis [53]; therefore, the calculated *p* values are interpreted as descriptive, not hypothesis testing.

## 3. Results

### 3.1. Neurotrophic Factors Were Differentially Expressed in Dog Bladder Tissues

In a smaller initial study, we compared the levels of the neurotrophic factor-related genes in the bladder mucosa and smooth muscle between two groups, Control and Decentralized, with a housekeeping gene, *β-Actin*, as the reference gene. The mRNA levels of the six tested genes: *BDNF*, *NGF*, *NT-3*, *GDNF*, *CNTF*, and hepatocyte growth factor (*HGF*) showed a gene x tissue effect (*p* = 0.01) and trended towards a gene effect (*p* = 0.07) and surgical group effect (*p* = 0.07). Supplemental Figure S2 shows the graphs and post hoc analysis results. *NT-3* mRNA expression was lower, and *BDNF* mRNA expression trended towards being lower, in the muscles of Decentralized bladders, versus Controls (*p* = 0.02 and *p* = 0.07, respectively). There were no other significant differences between the surgical groups for any of the other genes tested. In addition, in Control bladders, *BDNF* and *NT-3* mRNA expressions were higher in muscle, compared to mucosa (*p* = 0.04 and *p* = 0.03, respectively), while *HGF* levels were higher in the mucosa, compared to muscle (*p* = 0.005). These findings helped inform our protein expression studies, described next.

### 3.2. Enhanced BDNF Protein in Mucosa of ObNT-Reinn Bladders, and Reduced NT-3 Protein in Mucosa of ObNT-Reinn and Decentralized Bladders

**Figure 2:**
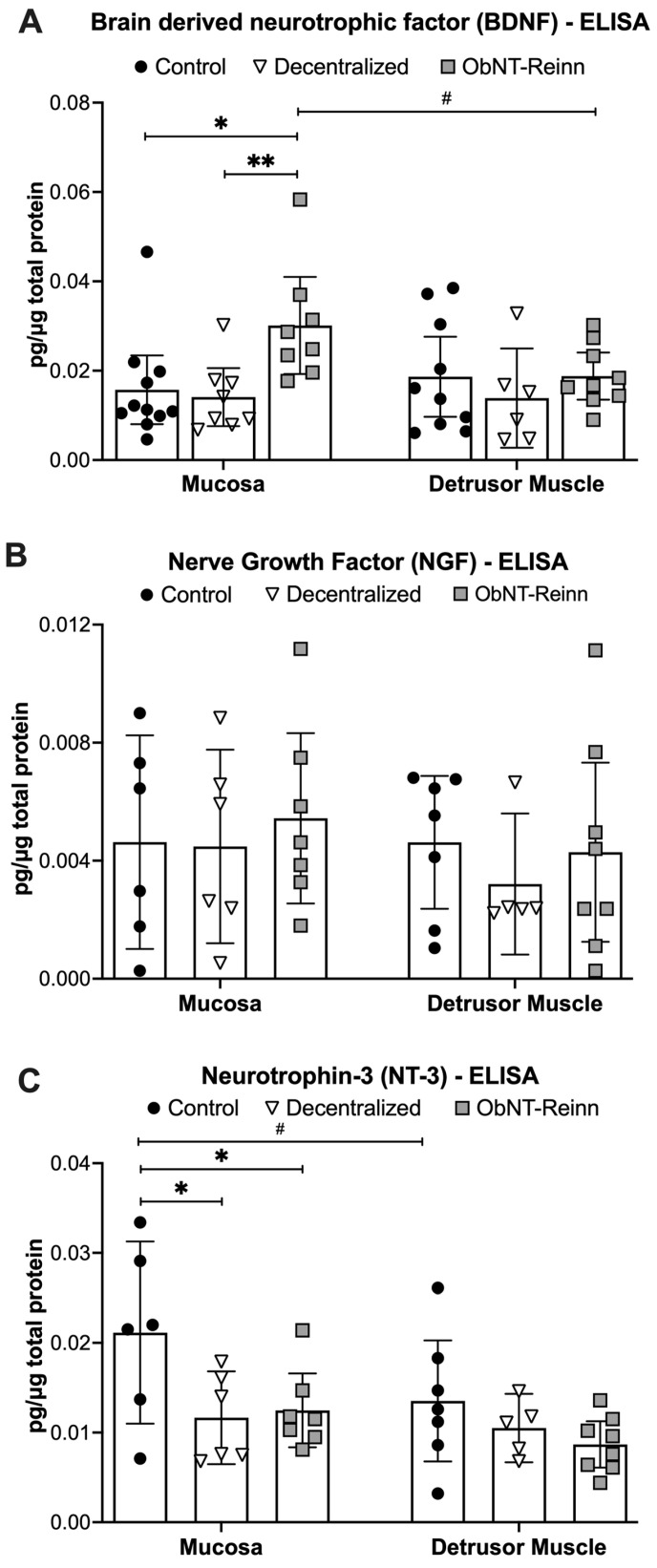
BDNF, NGF and NT-3 protein concentrations, measured by ELISA. BDNF (**A**) and NGF (**B**), and NT-3 (**C**) levels in mucosa and smooth muscle lysates of Control, Decentralized, and ObNT-Reinn bladders. The number of animals assayed per group were: 6–11 Control, 5–8 Decentralized, and 7–9 ObNT-Reinn. Shown are the means ± 95% CI. *: *p* < 0.05 and **: *p* < 0.01, compared within the same layer and between groups as shown. #: *p* < 0.05, compared between bladder mucosa and muscle layers as shown. Data were analyzed using repeated-measures, mixed-effects, and REM models, followed by Tukey’s multiple comparisons post hoc test.

Protein levels of BDNF showed a group effect (*p* = 0.02) in the mixed-effects model. Post hoc analyses showed that BDNF protein levels were ~2.0- to 2.2-fold higher in ObNT-Reinn mucosa, compared to Control and Decentralized mucosa (*p* = 0.01 and *p* = 0.008, respectively, Figure 2A). The mucosa of Decentralized and Control bladders showed similar BDNF levels, as did muscle across the three groups (Figure 2A). Also, BDNF protein levels were ~1.7-fold higher in mucosa versus muscle of ObNT-Reinn bladders (*p* = 0.03).

The mixed-effects model analysis of NGF protein levels showed no group, surgical, or interaction effects (Figure 2B). Mucosal and muscle levels were also similar.

Protein levels of NT-3 differed, showing group and tissue type effects in the mixed-effects model analysis (group effect, *p* = 0.009; tissue type effect, *p* = 0.03). Post hoc analyses showed that NT-3 protein levels were ~1.7- to 1.8-fold lower in ObNT-Reinn and Decentralized mucosa, respectively, compared to the Control mucosa (*p* = 0.03 and *p* = 0.02, respectively, Figure 2C). Also, NT-3 protein levels were ~1.7-fold higher in mucosa versus muscle in Control bladders (*p* = 0.02). NT-3 protein levels in the muscle were comparable between the groups.

### 3.3. Reduced Artemin Levels in the Mucosa of Decentralized and ObNT-Reinn Bladders, and Reduced GDNF Protein in Mucosa of Decentralized Bladders

**Figure 3:**
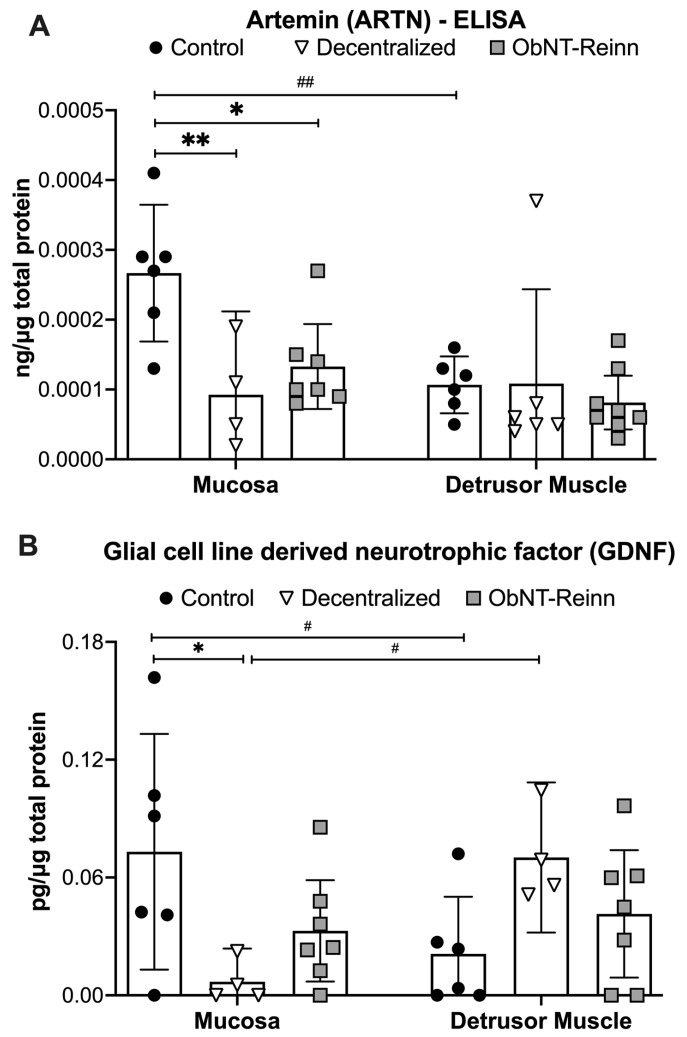
Artemin (ARTN) and GDNF protein concentrations, measured by ELISA. Artemin (**A**) and GDNF (**B**) levels in mucosa and smooth muscle lysates of Control, Decentralized, and ObNT-Reinn bladders. The number of animals assayed per group were: 6 Control, 4–6 Decentralized, and 7–8 ObNT-Reinn. Shown are means ± 95% CI. *: *p* < 0.05 and **: *p* < 0.01, compared within the same layer and between groups as shown. #: *p* < 0.05 and ##: *p* < 0.01, compared between bladder mucosa and muscle layers as shown. Data were analyzed using repeated-measures, mixed-effects, REM models followed by Tukey’s multiple comparisons post hoc test.

Protein levels of Artemin (ARTN) showed several differences in the mixed-effects model (group effect, *p* = 0.03; tissue type effect, *p* = 0.03; tissue type x group effect, *p* = 0.03). Post hoc analyses showed that ARTN levels were ~2- to 3-fold lower in ObNT-Reinn and Decentralized bladder mucosa, compared to Control bladder mucosa (*p* = 0.01 and *p* = 0.005, respectively, Figure 3A). ARTN levels in the muscle were comparable between the three groups. ARTN was ~2.5-fold higher in the mucosa versus muscle in Control bladders (*p* = 0.001).

Protein levels of GDNF showed a tissue type x group effect in the mixed-effects model (*p* = 0.004). Post hoc analyses showed that GDNF protein levels were ~10-fold lower in Decentralized mucosa, compared to Control mucosa (*p* = 0.02, Figure 3B). In Control bladders, GDNF protein levels were ~3.5-fold higher in the mucosa versus muscle layers (*p* = 0.01), while in Decentralized bladders, GDNF level was ~10-fold higher in the muscle versus mucosa (*p* = 0.02).

### 3.4. Similar CNTF Levels in Bladders of the Three Dog Groups

**Figure 4:**
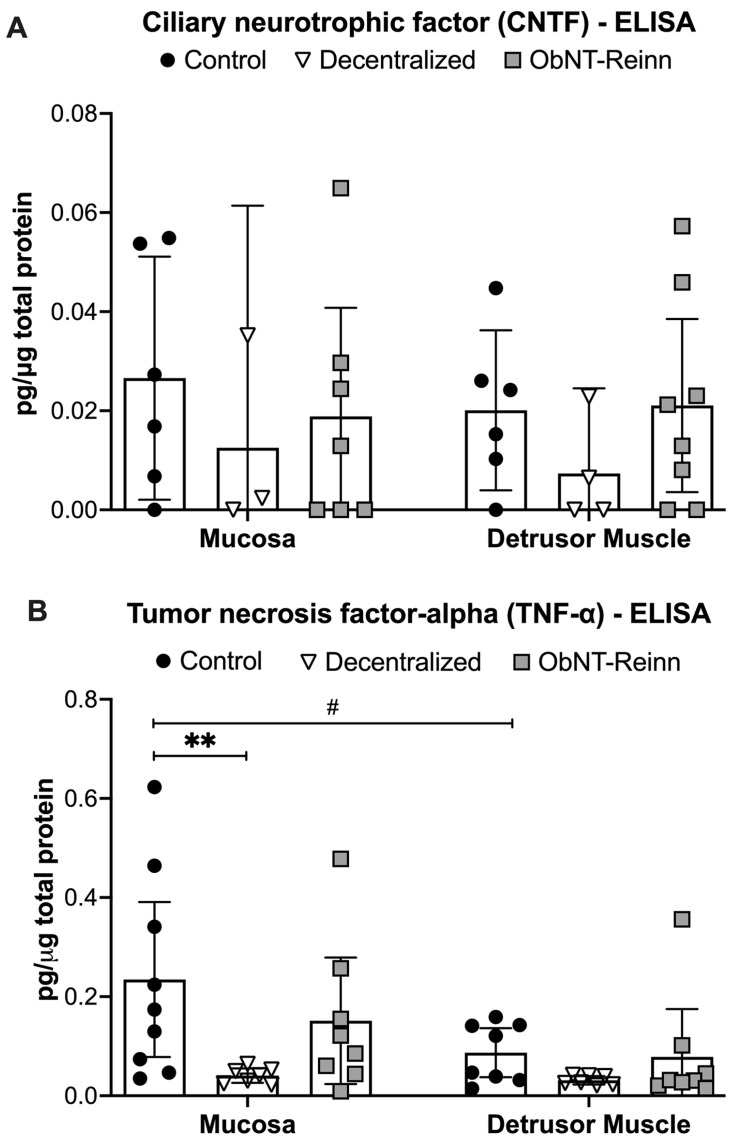
Protein concentrations of neuropoietic/neurodifferentiation cytokines, measured by ELISA. (**A**) Ciliary neurotrophic factor (CNTF) and (**B**) Tumor necrosis factor alpha (TNF-α) levels in mucosa and smooth muscle lysates of Control, Decentralized, and ObNT-Reinn bladders. The number of animals assayed per group were: 6–9 Control, 3–7 Decentralized, and 7–8 ObNT-Reinn. Means ± 95% CI are shown. **: *p* < 0.01, compared within the same layer and between groups as shown. #: *p* < 0.05, compared between bladder mucosa and muscle layers as shown. Data were analyzed using repeated-measures, mixed-effects, REM models followed by Tukey’s multiple comparisons post hoc test.

Mucosal and muscle expression levels of CNTF were similar between the dog bladders, as were mucosal versus muscle levels (Figure 4A).

### 3.5. Reduced TNF-α Levels in the Mucosa of Decentralized Bladders

Levels of the pro-inflammatory cytokine TNF-α showed a group effect (*p* = 0.01) in the mixed-effects model. Post hoc analyses showed that TNF-α protein levels were lower in Decentralized mucosa, compared to Control mucosa (~4.1-fold, *p* = 0.004, Figure 4B). TNF-α levels were back to Control levels in ObNT-Reinn mucosa. TNF-α levels were ~2- to 2.3-fold higher in mucosa versus muscle of Control bladders (*p* = 0.03).

### 3.6. Enhanced ROS Production in the Mucosa of ObNT-Reinn Bladders

**Figure 5:**
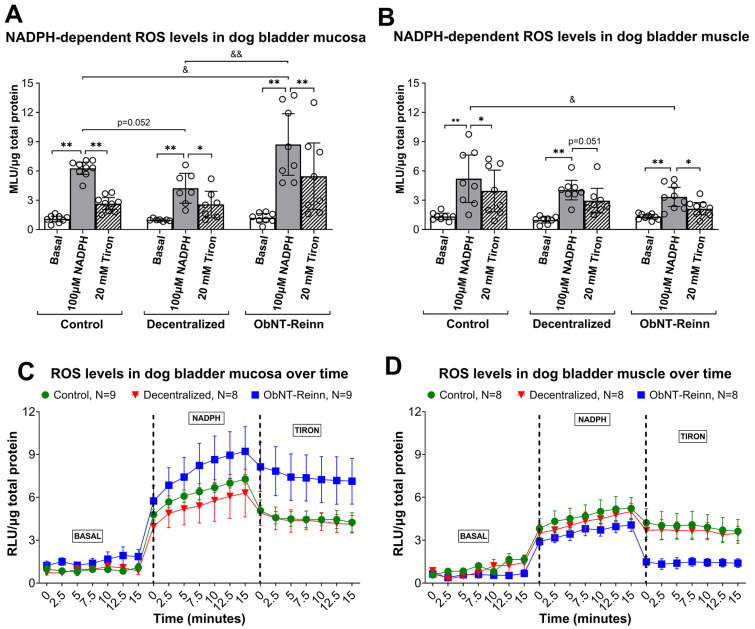
NADPH-dependent ROS levels assayed using lucigenin-enhanced chemiluminescence in mucosa and smooth muscle tissues of bladders from Control, Decentralized, and ObNT-Reinn animals. Total homogenates of mucosa (**A**) and muscle (**B**) were exposed to dark-adapted lucigenin in balanced salt solution and baseline was measured (Basal). The addition of NADPH (100 µM) enhanced superoxide production (**C**,**D**). This production was attenuated by the addition of 20 mM Tiron. Representative photon emission in response to the three different conditions (Basal, addition of NADPH, and addition of Tiron) are shown (**C**,**D**). MLU = mean luminescence units. RLU = relative luminescence units. The number of animals assayed per group were: eight Control, seven Decentralized, eight ObNT-Reinn. Data are presented as mean ± 95% CI. *: *p* < 0.05 and **: *p* < 0.01, comparing NADPH versus baseline or Tiron in each group. &: *p* < 0.05 and &&: *p* < 0.01, comparing NADPH between the three groups. Data were analyzed using repeated-measures, mixed-effects, REM models followed by Fisher’s LSD post hoc test.

Dog bladder mucosa and muscle samples were prepared as total homogenates for lucigenin-enhanced chemiluminescence assays. In each group and tissue type, the addition of 100 µM of NADPH to the buffer enhanced the lucigenin signal above background levels by stimulating ROS production (*p* ≤ 0.0001, Figure 5A–D). The mucosa of ObNT-Reinn bladders showed ROS levels that were ~1.4- to 2.1-fold higher than that in the Control (*p* = 0.02) and Decentralized (*p* = 0.0001) bladders, respectively (Figure 5A,C). Mucosal ROS levels trended towards being lower in Decentralized bladders, compared to Controls (1.5-fold, *p* = 0.052, Figure 5A). However, ROS levels in the muscle layers were decreased by 1.6-fold in the ObNT-Reinn bladders, compared to Control bladders (*p* = 0.01, Figure 5B,D).

ROS levels in response to the superoxide scavenger, Tiron (20 mM), were significantly lower than those elicited by NADPH (Figure 5A–D) in the bladder mucosa (1.6- to 2.4-fold, Figure 5A,C) and muscle (1.3- to 1.7-fold, Figure 5B,D), suggesting that NADPH oxidase enzyme (NOX) is a significant source of superoxide in dog bladder mucosa and muscle in response to NADPH exposure.

### 3.7. Localization of BDNF and TRK B in Bladder Wall Using Immunohistochemistry

**Figure 6:**
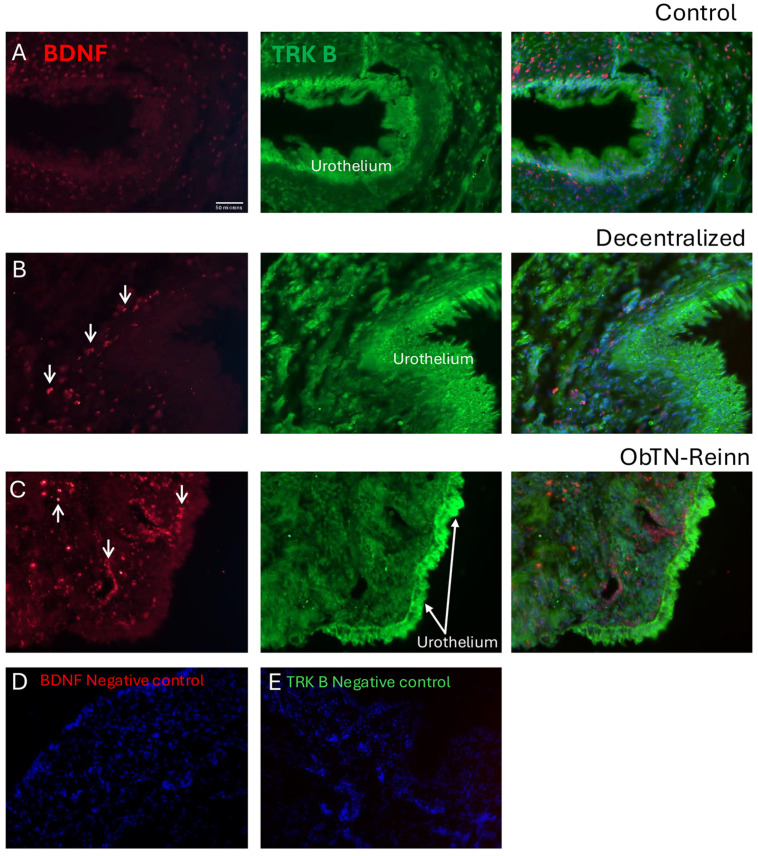
Immunoexpression of BDNF (red) and its receptor (TRK B) in the mucosal layer of Control (**A**), Decentralized (**B**), and (**C**) ObNT-Reinn bladders. Left panels of (**A**–**C**) show BDNF immunoexpression. Middle panels of (**A**–**C**) show TRK B immunoexpression. Right panels of (**A**–**C**) show merged images of BDNF and TRK B, and DAPI counterstaining. Arrows in left panels of (**B**,**C**) indicate a few of the BDNF+ immune-like cells. Urothelium is indicated in each panel. (**D**,**E**) Negative control images in which only secondary antibodies were used (the primary antibodies were omitted). Scale bar in left panel (**A**) (50 microns) applies to all other panels.

**Figure 7:**
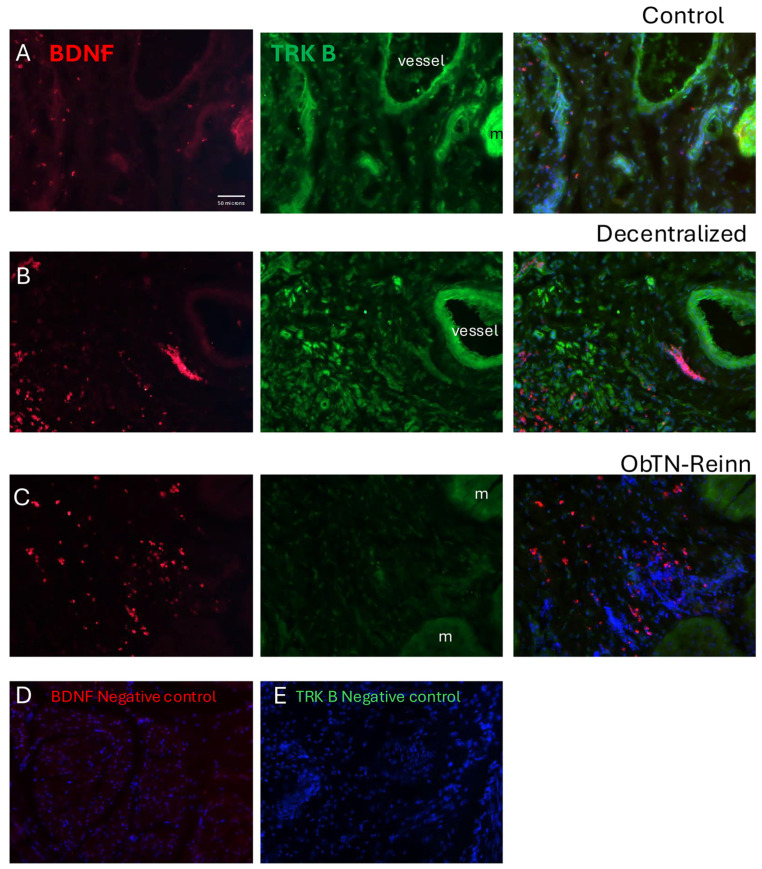
Immunoexpression of BDNF (red) and its receptor (TRK B) in the submucosal layer of Control (**A**), Decentralized (**B**), and (**C**) ObNT-Reinn bladders. Left panels of (**A**–**C**) show BDNF immunoexpression. Middle panels of (**A**–**C**) show TRK B immunoexpression. Right panels of (**A**–**C**) show merged images of BDNF and TRK B, and DAPI counterstaining. Vessels and muscle (m) are indicated in each panel when present. (**D**,**E**) Negative control images in which only secondary antibodies were used (the primary antibodies were omitted). Scale bar in left panel (**A**) (50 microns) applies to all other panels.

**Figure 8:**
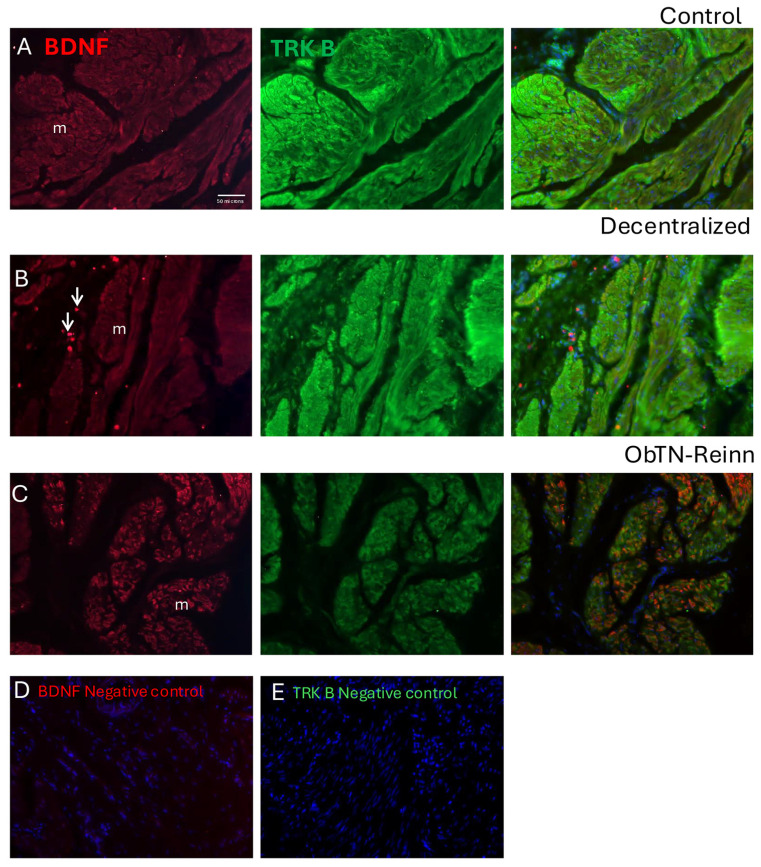
Immunoexpression of BDNF (red) and its receptor (TRK B) in the detrusor muscle layer of Control (**A**), Decentralized (**B**), and (**C**) ObNT-Reinn bladders. Left panels of (**A**–**C**) show BDNF immunoexpression. Middle panels of (**A**–**C**) show TRK B immunoexpression. Right panels of (**A**–**C**) show merged images of BDNF and TRK B, and DAPI counterstaining. Arrows in left panels of (**B**) indicate a few of the BDNF+ immune-like cells; m = muscle. (**D**,**E**) Negative control images in which only secondary antibodies were used (the primary antibodies were omitted). Scale bar in left panel (**A**) (50 microns) applies to all other panels.

**Figure 9:**
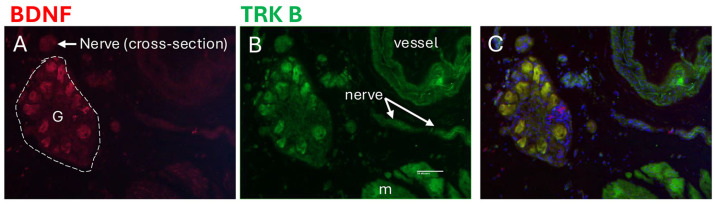
Immunoexpression of BDNF (red) and its receptor (TRK B) in an intramural ganglion from an ObNT-Reinn bladder. (**A**) BDNF immunoexpression. (**B**) TRK B immunoexpression. (**C**) Merged images of BDNF and TRK B, and DAPI counterstaining. G = ganglion; nerve profiles indicated in cross-section (**panel A**) and longitudinal section (**panel B**; m = muscle). Scale bar in left panel (**B**) (50 microns) applies to all other panels.

Immunohistochemistry was used to show the localization of BDNF and the TRK B (a BDNF receptor) in the mucosa, submucosa, detrusor muscle, intramural ganglia, and nerves within the bladder walls (Figure 6, Figure 7, Figure 8 and Figure 9).

In Control bladder mucosa (Figure 6A), BDNF immunoexpression was seen in a small subset of myofibroblast-like cells located in the connective tissues of this layer (Figure 6A left panel). TRK B immunoexpression was seen in several cell types, including urothelial and myofibroblast-like cells (including the BDNF+ myofibroblast-like cells) (Figure 6A middle and merged-image right panels). In Decentralized mucosa (Figure 6B left panel), immune-like cells in the connective tissues were BDNF immunopositive. TRK B immunostaining was similar to Control bladders (including in the urothelium when present; Figure 6B middle panel). In ObNT-Reinn mucosa (Figure 6C), more BDNF immunoexpression was observed relative to the other groups, including in many small immune-like and myofibroblast-like cells (Figure 6C left and right merged image panel). TRK B immunostaining was similar to the other groups (Figure 6C middle and right panels). There was no co-localization between BDNF and TRK B in the immune-like cells. The negative control sections in which only secondary antibodies were added (i.e., no primary antibodies) showed only the DAPI counterstain (Figure 6D,E).

In the Control submucosal layer (Figure 7A), BDNF immunoexpression was present in myofibroblast-like cells, and in muscle cells located in the adjacent detrusor layer (Figure 7A left panel). TRK B immunoexpression was observed in several cell types, including myofibroblast-like cells and endothelial cells of blood vessels (Figure 7A middle and right panels). In Decentralized submucosum (Figure 7B), BDNF immunoexpression was observed in small numbers of immune like cells (Figure 7B left panel). TRK B immunostaining was similar to Controls (Figure 7B middle panel). In ObNT-Reinn submucosum (Figure 7C), BDNF immunoexpression was observed in many immune like cells (Figure 7C left panel). TRK B was myofibroblast like cells and in muscle cells located in the adjacent detrusor layer (Figure 7C middle and right panels). There was no co-localization between BDNF and TRK B in the immune-like cells. The negative control sections in which only secondary antibodies were added (i.e., no primary antibodies) showed only the DAPI counterstain (Figure 7D,E).

In the Control detrusor muscle layer (Figure 8A), BDNF immunoexpression was observed in the smooth muscle cells of detrusor layer bundles (Figure 8A, left panel). High levels of TRK B immunoexpression were observed in these same muscles (Figure 8A middle and right panels). In the Decentralized detrusor muscle layer (Figure 8B), BDNF immunoexpression was also observed in immune cells, although less in the smooth muscle cells relative to Control bladders (Figure 8B, left panel). TRK B immunoexpression was similar to Control bladders (Figure 8B middle and right panels). In the ObNT-Reinn detrusor muscle layer (Figure 8C), BDNF immunoexpression in the smooth muscle cells was intensity as in Control bladders, although more heterogeneous in distribution (Figure 8C, left panel). TRK B immunoexpression was similar to Control bladders (Figure 8C middle and right panels). The smooth muscle cells showed co-localization between the punctate staining of the BDNF and intense staining of the TRK B in the smooth muscles (Figure 8A–C right panels). The negative control sections in which only secondary antibodies were added (i.e., no primary antibodies) showed only the DAPI counterstain (Figure 8D,E).

In neural tissue in the bladder wall (Figure 9), BDNF immunoexpression was seen in the cytoplasm of intramural ganglion neurons, yet only low levels in nerve profiles were shown in both cross-sectional and longitudinal sections (Figure 9A,C). TRK B immunoexpression was observed in the cytoplasm of these same neuronal cell bodies, as well as in nerve profiles and smooth muscle (Figure 9B,C). Co-localization immunostaining showed clear overlap between the BDNF and TRK B immunostaining in the cytoplasm of intramural ganglion neuronal cell bodies (Figure 9A–C).

### 3.8. DHE Detection of ROS

**Figure 10:**
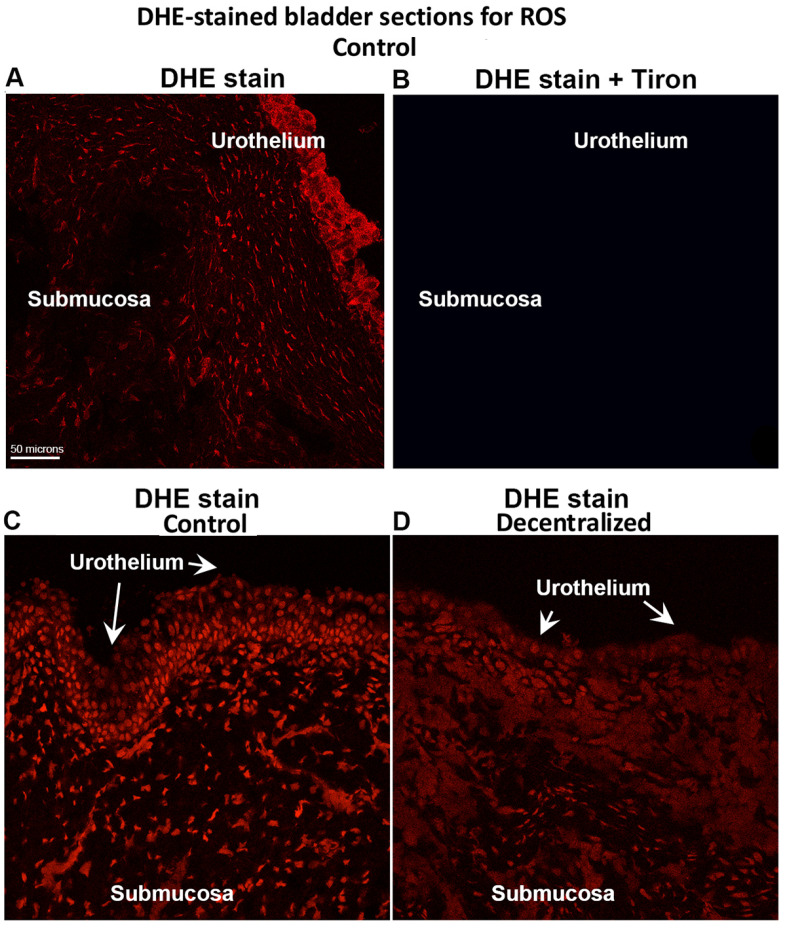
DHE staining of ROS in bladder tissue. (**A**) DHE staining in Control mucosa and submucosa. (**B**) Negative control of DHE staining of ROS generated by adding Tiron prior to adding DHE to tissue and subsequent imaging. (**C**) DHE staining in a different Control animal’s mucosa and submucosa. (**D**) DHE staining in a Decentralized animal, showing less staining than in Control animals. Confocal images. Scale bar in panel (**A**) (50 microns) applies to all other panels.

DHE staining was used to detect ROS. As shown in Figure 10, the urothelium, mucosa and submucosal layers contained many small cells that stained positive ROS in both Control and Decentralized bladders (Figure 10A,C,D). The addition of Tiron blocked the ROS staining (Figure 10B). Similarly to the data shown in Figure 5A, the ROS staining in Controls was brighter than in Decentralized animals.

### 3.9. Correlations Between Biomarker Levels

As shown in Table 2, there was a moderate positive correlation between BDNF levels in the mucosa versus muscle layers, and a strong positive correlation between TNF-α levels in the mucosa versus muscle layers. There was also a moderate negative correlation between GDNF levels in the mucosa versus muscle layers.

### 3.10. Correlations Between Biomarker Levels and Histological Outcomes

Levels of these analytes were correlated with previously published histological findings from the bladder wall of these same animals [22]—data are shown in Supplemental Figure S3A–E. The upper part of Table 3 shows that mucosal levels of BDNF correlated moderately and positively with scores of inflammation in the submucosa and detrusor layers and detrusor muscle layer thickness, and strongly and positively with axonal density (specifically, with PGP9.5 immunostained axons, Supplemental Figure S3E). Mucosal levels of CNTF correlated strongly and negatively with urothelial integrity score. As shown in Table 4, mucosal levels of ROS correlated strongly and negatively with detrusor thickness and number of neuronal cell bodies in intramural ganglia, and moderately and negatively with inflammation scores in the full bladder wall thickness.

As shown in the lower part of Table 3, muscle levels of NT-3 correlated moderately and negatively with inflammation scores in the detrusor layer. Muscle levels of ARTN showed a trend towards a moderate and negative correlation with the axon density (*p* = 0.055). Muscle levels of TNF-α in the muscle correlated moderately and negatively with muscle layer thickness.

### 3.11. Correlations Between Biomarker Levels and Functional Outcomes

Additionally, levels of the biomarkers were correlated with previously published functional outcomes of these same animals [19]—data are shown in Supplemental Figure S3F,G. Specifically, the biomarkers were correlated with maximum bladder contractions: (1) after electrical stimulation of the vesical nerve near the bladder wall (Controls and Decentralized animals) or transferred obturator nerve, on the spinal cord side of the coaptation site (ObNT-Reinn animals), or (2) after electrical stimulation of the spinal roots of origin of these peripheral nerves (which is L7-S3 for the vesical nerves, and stimulated in Controls and Decentralized animals, or L2-L6 for the transferred obturator nerve, and stimulated in ObNT-Reinn animals). As shown in Table 5, mucosal levels of GDNF, TNF-α and ROS correlated moderately to strongly with functional electrical stimulation of the vesical or transferred obturator nerves. In addition, mucosal levels of ROS correlated strongly with functional electrical stimulation of the spinal root of origin of the vesical or transferred obturator nerves. Also shown in Table 5, muscle levels of BDNF and GDNF correlated moderately or strongly with functional electrical stimulation of the vesical or transferred obturator nerves, as did muscle levels of TNF-α (Table 5).

### 3.12. Correlations Between Biomarker Levels and Age of Animals

Levels of analytes were also correlated with the animal’s age (Table 6). Mucosal levels of NGF correlated moderately and negatively with age. Muscle levels of NT-3 correlated moderately and positively with their age, while muscle levels of ROS showed a moderate and negative correlation with their age.

## 4. Discussion

We sought to examine the expression of several neurotrophic factors, TNF-α, and ROS production in the mucosa and muscle tissues of dog bladders that had undergone long-term decentralization, or decentralization followed by reinnervation, compared to Control bladders. In an initial pilot study, examination of mRNA levels of neurotrophins showed only reduced NT-3 and BDNF expression in Decentralized bladder muscle, compared to Control bladder muscle, but no between-group differences in the mucosa. This differed from the protein analyses that showed mainly group differences in this layer. BDNF protein levels were higher in ObNT-Reinn mucosa, compared to other groups, as were ROS levels, and in ObNT-Reinn mucosa versus muscle, perhaps due to the reinnervation of the bladder wall by a somatic nerve (the obturator) that serves as the exogenous source of these factors. GDNF and TNF-α protein levels were lower in Decentralized mucosa, versus Control mucosa, perhaps due to the prolonged decentralization—although rescued by the nerve transfer strategy since GDNF was back to Control levels in the ObNT-Reinn mucosa. NT-3 and ARTN protein levels were lower in ObNT-Reinn and Decentralized mucosa, versus Control mucosa, again, perhaps due to the prolonged decentralization. ROS levels were lower in the ObNT-Reinn muscle, compared to Control muscle. Previously published histological findings from the bladder wall of these same animals were used in correlational analyses [22] (see Supplemental Figure S3A–E). Urothelial integrity correlated with mucosal CNTF, scores of inflammation in the submucosal layer correlated with mucosal BDNF and TNF-α levels, detrusor muscle thickness correlated with mucosal BDNF and ROS and with muscle TNF-α, while scores of inflammation in the detrusor layer correlated with mucosal BDNF and muscle NT-3. From a neuroanatomical standpoint, the number of neuronal cell bodies in intramural ganglia correlated moderately and positively with mucosal levels of BDNF and CNTF, and bladder axon density correlated strongly and positively with mucosal BDNF. In addition, mucosal levels of GDNF, TNF-α and ROS correlated with functional electrical stimulation of the vesical or transferred obturator nerve, mucosal levels of ROS correlated strongly with functional electrical stimulation of the spinal root of origin of the vesical or transferred obturator nerves, and muscle levels of BDNF, GDNF and TNF-α correlated with functional electrical stimulation of the vesical or transferred obturator nerve. Yet, most intriguing was the elevation of BDNF, GDNF and TNF-α in the mucosa of ObNT-Reinn bladder (BDNF was higher than the other groups, while GDNF and TNF-α resolved back to Control levels), perhaps due to the regrowth of now somatic axons into the bladder from the obturator nerve after transfer to this autonomic end organ.

BDNF was chosen as one candidate of investigation because it is secreted by bladder tissues at higher levels in individuals with detrusor underactivity (DU) with more functional recovery, versus individuals with DU and no functional recovery [54,55]. Our observed BDNF expression in the mucosa and detrusor smooth muscle layers of Control dog bladders (Figure 2A and Table 2) is in line with reported expression of BDNF in bladder urothelium and smooth muscle layers [54,56,57,58,59,60]. The differences between mRNA and protein expression was anticipated since BDNF has a paracrine feature and able to diffuse, suggesting that the site of expression of BDNF is not necessarily the same as its site of action [61]. The enhanced BDNF expression levels in the mucosa of ObNT-Reinn bladders (Figure 2A) may be due to new somatic neuronal pathway created by the reinnervation strategy that facilitated a return of bladder function after decentralization [19]. This is supported by the strong correlation between mucosal BDNF and increased axon density in ObNT-Reinn bladders (Table 3), and muscle BDNF with bladder contraction after functional electrical stimulation of the vesical or transferred obturator nerves (Table 5), with correlations performed using previously reported data [19,22] (see Supplemental Figure S3). Our data are consistent with a rat study in which BDNF is upregulated following nerve transection and repair, with a delay in that upregulation until improved functional neuronal recovery had occurred [62]. A murine study demonstrated that BDNF knockout mice exhibit severe neuronal innervation deficiency in the bladder [56]. Trophic proteins play an important role in regulating cell growth, differentiation, migration, survival and neurite outgrowth in the nervous system under both healthy and pathological conditions [63]. BDNF, specifically, plays a functional regulation of motor and sensory neurons in the intact peripheral nervous system [64,65], mediates neuronal function under pathological conditions [64,66,67], and upregulates soon after injury [38,68,69]. In rodents, endogenously produced BDNF is necessary for axonal regeneration following nerve injury [70]. Following spinal cord injury, administration of BDNF protects bladder function by reducing denervation and preserving nerves [71]. Also, in a rat model of inflammation, BDNF and other neurotrophins increase shortly after an inflammatory stimulus in visceral tissues, including bladder, suggestive of a role in inflammation [57].

Regarding GDNF, its receptors up-regulate after injury in sacral spinal parasympathetic preganglionic neurons [72]. The lower expression of GDNF protein in the Decentralized mucosa versus Control mucosa (Figure 3B) is consistent with severe denervation models in which GDNF expression is upregulated in injured nerves early post-injury, but down-regulated with continued denervation [73,74,75]. The recovered expression of GDNF in the ObNT-Reinn mucosa could suggest a potential role for GDNF in promoting neurite survival, or mediating neuroplasticity, as was previously reported in many types of neurons including peripheral autonomic, sensory and motor [76,77,78,79].

NT-3 supports the growth and survival of sympathetic and sensory neurons in the peripheral nervous system [80], and stimulates neurite outgrowth in rat pelvic ganglia [81]. A neuroprotective effect of NT-3 is observed only during the acute phase of damage [82]. Therefore, we anticipated its lowered levels in the mcosa of ObNT-Reinn and Decentralized bladders (Figure 2C), which are undergoing chronic injury processes [83]. That may explain the negative correlation between NT-3 and muscle inflammation (Table 3). The similar levels of NT-3 in the muscle layers across the groups agrees with a report of unchanged expression of NT-3 mRNA in skeletal muscle after nerve transection [84,85].

ARTN promotes the survival of bladder motor neurons in mouse lumbar spinal cord [86,87], in vitro survival of rat sensory and sympathetic neurons, and peripheral nerve homoeostasis [87,88,89,90,91]. Expression levels of ARTN are relatively low in many peripheral adult tissues, and comes mainly from Schwann cells [87]. ARTN upregulates after nerve injury and selectively binds the GDNF family receptor, GFRα3, whose expression is highly restricted to sensory neurons [87]. Damaged sensory neurons exhibit enhanced sensitivity to ARTN [92]. We speculate that the decreased ARTN expression in Decentralized and ObNT-Reinn bladder mucosa is a result of the prolonged extensive sensory denervation, similar to findings from another model [88], or because the ability of neurons to survive (e.g., intramural ganglionic neurons), capacity of Schwann cells to support neuronal regeneration, or trophic factor production have decreased over time, as reported [90,91].

Different neurotrophins promote nerve regeneration via different mechanisms [84,93,94]. The regulation of neurotrophic factor expression may differ with different types of nerve injuries [94,95], or may have selective dependence [96,97]. The selective expression of neurotrophic factors correlates with a preferential reinnervation of proper neuronal pathways [98]. The lower protein levels of NT-3 and ARTN in the mucosa of Decentralized and ObNT-Reinn bladders could reflect lower protein secretion or higher ligand consumption in these groups [80]. There may also be post-translational modifications affecting final levels [94,95,99,100]. Further investigation is necessary to help in understanding those mechanisms.

TNF-α is both a mediator of peripheral inflammation and a neurotrophic factor during neuroregenerative processes [101]. After transection of sacral ventral roots in our dog model, we have observed neuroinflammation and reduced motor inputs to the bladder, which could enhance the release of neurochemicals from axon terminals into the bladder, as shown in a primate model of cauda equina injury [22,31,102]. Here, we observed decreased TNF-α in Decentralized bladder mucosa (Figure 4B). TNF-α might be transiently increased after decentralization to initiate the pro-inflammatory response to injury [103,104,105,106,107,108]. However, with time, the Decentralized animals have recurrent bacteriuria that require repeated antibiotic treatments until study end [22,50]. Such chronic inflammation probably disrupted the urothelial integrity, and enhanced inflammation in underlying bladder layers [1,109,110]. The continued antibiotic treatments provided to this group may also disrupt beneficial urothelial bacteria (urobiota), as reported in human intestines [111]. Maintenance of urothelial integrity is critical in promoting proper protection against infections [112,113]. We speculate that disruption of the urothelial layer using antibiotics resulted in reduced TNF-α levels and consequently, a lower defense against infections [111].

In contrast, the observed increase in TNF-α in the mucosa of ObNT-Reinn bladders to close to the Control level is suggestive of tissue recovery, as suggested by other studies [108,114,115]. In support, we found that TNF-α levels was similar in ObNT-Reinn bladders to that observed in Control bladders. Also supporting a new neuronal pathway hypothesis, TNF-α in the muscle correlated strongly and positively with the functional electrical stimulation outcomes shown in Table 5.

Neurotrophins’ signaling may also involve ROS activity [47]. The main species and most stable form of ROS, hydrogen peroxide (H_2_O_2_, 100 µM), has been shown to induce bladder muscle contractions, and NOX is the main source of a relatively unstable and short-lived ROS, superoxide [52]. We have previously shown increased NADPH-dependent ROS levels in normal dog bladders, specifically in the mucosa, suggesting that mucosa is the main source for Nox enzymes [52]. It is well documented that ROS promote injury-induced axonal regeneration, enhance neurite outgrowth and modulate neuronal plasticity centrally and peripherally [116,117,118,119,120,121]. In this study, ROS levels correlated with detrusor layer thickness and inflammation in the detrusor layer, and with the functional electrical stimulation outcomes shown in Table 5. BDNF and ROS may contribute to remodeling processes, with BDNF-induced ROS generation via Nox activation are strictly required for the dendritic spine remodeling [46,122,123].

## 5. Conclusions

Our findings of enhanced levels of BDNF and restored levels of GDNF in reinnervated bladder mucosa after peripheral nerve injury and surgical repair suggest that both may contribute, at least in part, to promoting the reestablishment of bladder innervation after nerve injury and surgical repair in dogs. The strong correlations between BDNF and axon density (r = 0.76) and the detrusor layer thickness (r = 0.56) suggest that the exogenous applications of BDNF into the bladder wall following decentralization and surgical rerouting are further supportive. Keeping in mind that our results are biochemical assays and therefore are limited in their significance. Further studies are needed, such as the application of exogenous BDNF (or GDNF), to determine whether either is suitable for promoting improved appropriate functional recovery, as previously reported in a rat model in which exogenous BDNF promoted facial nerve recovery after injury [124]. Other factors that are highly relevant for the repair process, e.g., vascular endothelial growth factor (VEGF) or insulin growth factor 1 (IGF1) [125,126] should also be explored.

## Figures and Tables

**Table 1:** Gene specific qPCR information.

Gene	Assay	Reporter Dye
*ACTB (b-Actin)*	Cf04931159	FAM
*BDNF*	Cf02718934	FAM
*NTF3*	Cf02700489	FAM
*GDNF*	Cf02691052	FAM
*CNTF*	Cf03460095	FAM
*HGF (hepatocyte growth factor)*	Cf02692661	FAM
*NGF*	Cf02697134	FAM

**Table 2:** Correlation of neurotrophic factors in bladder tissues. Bolded letters indicate significant findings here and in subsequent tables. *: *p* < 0.05 and **: *p* < 0.01, significant correlation.

Neurotrophin	BDNF	NGF	NT-3	ARTN	GDNF	CNTF	TNF-α
Mucosa vs. muscle	**r = 0.4,** *p* = 0.04 *	r = −0.25, *p* = 0.36	r = 0.2, *p* = 0.5	r = 0.13, *p* = 0.62	**r = −0.5,** *p* = 0.046 *	r = −0.04, *p* = 0.7	**r = 0.56,** *p* = 0.005 **

**Table 3:** Correlation between neurotrophic factors and histological changes in bladder. Bolded letters indicate significant findings here and in subsequent tables. *: *p* < 0.05 and **: *p* < 0.01, significant correlation.

Histological Parameters	Urothelial Integrity Score (0 = Normal, 3 = Detached)	Submucosa, Inflammation Score (0 = Normal, 3 = Abnormal)	Detrusor Layer Thickness (mm)	Detrusor Layer, Inflammation Score (0 = Normal, 3 = Abnormal)	#Neuronal Cell Bodies/ Ganglion (#/mm^2^)	Axon Density (#/mm^2^)
**Mucosa**						
BDNF	r = −0.31, *p* = 0.13	**r = 0.46, ** *p* = 0.03 *	**r = 0.56, ** *p* = 0.03 *	**r = 0.46, ** *p* = 0.03 *	**r = 0.43, ** *p* = 0.04 *	**r = 0.76,** *p* < 0.0001 **
NGF	r = 0.03, *p* = 0.46	r = 0.30, *p* = 0.17	r = 0.0002, *p* = 0.5	r = 0.3, *p* = 0.2	r = −0.08, *p* = 0.41	r = 0.31, *p* = 0.2
NT-3	r = −0.17, *p* = 0.3	r = 0.30, *p* = 0.17	r = 0.11, *p* = 0.4	r = −0.3, *p* = 0.2	r = −0.17, *p* = 0.33	r = 0.3, *p* = 0.2
ARTN	r = −0.24, *p* = 0.28	r = −0.43, *p* = 0.08	r = 0.01, *p* = 0.5	r = −0.5, *p* = 0.09	r = −0.23, *p* = 0.27	r = 0.46, *p* = 0.11
GDNF	r = 0.23, *p* = 0.27	r = −0.37, *p* = 0.15	r = 0.15, *p* = 0.35	r = −0.3, *p* = 0.15	r = −0.3, *p* = 0.2	r = 0.04, *p* = 0.45
CNTF	**r = −0.71, ** *p* = 0.02 *	r = 0.23, *p* = 0.26	r = 0.59, *p* = 0.06	r = 0.23, *p* = 0.3	**r = 0.6, ** *p* = 0.04 *	r = 0.4, *p* = 0.14
TNF-α	r = −0.12, *p* = 0.33	r = −0.04, *p* = 0.43	r = −0.04, *p* = 0.45	r = − 0.04, *p* = 0.4	r = −0.07, *p* = 0.4	r = 0.02, *p* = 0.5
**Muscle**						
BDNF	r = −0.004, *p* = 0.5	r = 0.12, *p* = 0.32	r = −0.14, *p* = 0.3	r = 0.12, *p* = 0.32	r = 0.08, *p* = 0.4	r = 0.3, *p* = 0.13
NGF	r = 0.2, *p* = 0.3	r = −0.32, *p* = 0.10	r = −0.4, *p* = 0.1	r = −0.32, *p* = 0.12	r = −0.26, *p* = 0.2	r = −0.2, *p* = 0.3
NT-3	r = 0.12, *p* = 0.4	r = −0.32, *p* = 0.12	r = 0.12, *p* = 0.4	**r = −0.53, ** *p* = 0.04 *	r = −0.3, *p* = 0.2	r = −0.15, *p* = 0.3
ARTN	r = 0.14, *p* = 0.3	r = −0.37, *p* = 0.12	r = −0.006, *p* = 0.5	r = −0.4, *p* = 0.12	r = −0.4, *p* = 0.14	r = −0.53, *p* = 0.055
GDNF	r = −0.15, *p* = 0.36	r = 0.37, *p* = 0.13	r = 0.05, *p* = 0.45	r = 0.4, *p* = 0.13	r = 0.35, *p* = 0.2	r = −0.1, *p* = 0.4
CNTF	r = 0.3, *p* = 0.3	r = 0.13, *p* = 0.36	r = 0.1, *p* = 0.41	r = 0.13, *p* = 0.4	r = −0.2, *p* = 0.33	r = 0.16, *p* = 0.3
TNF-α	r = −0.01, *p* = 0.5	r = −0.18, *p* = 0.25	**r = −0.5,** *p* = 0.046 *	r = −0.2, *p* = 0.25	r = −0.11, *p* = 0.3	r = −0.2, *p* = 0.3

**Table 4:** Correlation between ROS levels and histological changes in bladder tissues. Bolded letters indicate significant findings here and in subsequent tables. *: *p* < 0.05, significant correlation.

Histological Parameters	Urothelial Integrity Score (0 = Normal, 3 = Detached)	Submucosa, Inflammation Score (0 = Normal, 3 = Abnormal)	Detrusor Layer Thickness (mm)	Detrusor Layer, Inflammation Score (0 = Normal, 3 = Abnormal)	# Neuronal Cell Bodies/ Ganglion (#/mm^2^)	Axon Density (#/mm^2^)
**Mucosa**	r = 0.26, *p* = 0.22	r = 0.07, *p* = 0.40	**r = −0.67, ** *p* = 0.02 *	**r = −0.44, ** *p* = 0.04 *	r = 0.11, *p* = 0.4	r = 0.3, *p* = 0.2
**Muscle**	r = 0.001, *p* = 0.5	r = −0.02, *p* = 0.46	r = −0.26, *p* = 0.25	r = −0.4, *p* = 0.08	r = 0.03, *p* = 0.5	r = 0.1, *p* = 0.4

**Table 5:** Correlation between biomarkers and functional outcomes. Bolded letters indicate significant findings here and in subsequent tables. *: *p* < 0.05, significant correlation.

Functional Outcome	Bladder Contraction After Electrical Stimulation of Vesical or Transferred Obturator Nerve	Bladder Contraction After Electrical Stimulation of Spinal Root of Origin (Vesical (L7-S3) or Transferred Obturator (L2-L6))
**Mucosa**		
BDNF	r = 0.31, *p* = 0.45	r = 0.09, *p* = 0.35
NGF	r = −0.03, *p* = 0.46	r = 0.07, *p* = 0.42
NT-3	r = 0.07, *p* = 0.37	r = 0.26, *p* = 0.24
ARTN	r = 0.44, *p* = 0.09	r = −0.15, *p* = 0.34
GDNF	r = 0.45, *p* = 0.04 *	r = 0.43, *p* = 0.08
CNTF	r = −0.31, *p* = 0.13	r = 0.13, *p* = 0.35
TNF-α	r = 0.76, *p* = 0.003 *	r = 0.34, *p* = 0.08
ROS	r = 0.56, *p* = 0.04 *	r = 0.74, *p* = 0.0005 *
**Muscle**		
BDNF	r = 0.40, *p* = 0.04 *	r = −0.03, *p* = 0.45
NGF	r = 0.36, *p* = 0.06	r = −0.22, *p* = 0.22
NT-3	r = 0.44, *p* = 0.09	r = −0.15, *p* = 0.32
ARTN	r = 0.46, *p* = 0.67	r = −0.37, *p* = 0.12
GDNF	r = −0.75, *p* = 0.04 *	r = −0.41, *p* = 0.11
CNTF	r = 0.28, *p* = 0.15	r = −0.02, *p* = 0.47
TNF-α	r = 0.73, *p* = 0.006 *	r = 0.16, *p* = 0.26
ROS	r = −0.29, *p* = 0.18	r = 0.10, *p* = 0.35

**Table 6:** Correlation between animal age and neurotrophic factors and ROS levels in bladder tissues. Bolded letters indicate significant findings here and in subsequent tables. *: *p* < 0.05, significant correlation.

Neurotrophin	BDNF	NGF	NT-3	ARTN	GDNF	CNTF	TNF-α	ROS
**Mucosa**	r = −0.04, *p* = 0.4	**r = −0.52,** *p* = 0.01 *	r = 0.1, *p* = 0.3	r = 0.1, *p* = 0.3	r = 0.2, *p* = 0.2	r = −0.1, *p* = 0.4	r = −0.3, *p* = 0.06	r = 0.003, *p* = 0.5
**Muscle**	r = 0.03, *p* = 0.47	r = 0.2, *p* = 0.2	**r = 0.46, ** *p* = 0.02 *	r = 0.1, *p* = 0.3	r = 0.1, *p* = 0.3	r = −0.26, *p* = 0.16	r = 0.04, *p* = 0.4	**r = −0.4, ** *p* = 0.04 *

## Data Availability

The original contributions presented in this study are included in the article/Supplementary Material. Further inquiries can be directed to the corresponding authors.

## Supplementary Materials

The following supporting information can be downloaded at: https://www.mdpi.com/article/10.3390/cells14060406/s1, Figure S1: Diagram of Surgeries Figure S2: Enhanced BDNF and ROS in mucosa of lower motor neuron-lesioned dog bladder following somatic motor nerve transfer. Figure S3: Histological and Functional Data Outcomes.
