# Supplementary material for: Enhanced BDNF and ROS in Mucosa of Lower Motor Neuron Lesioned Dog Bladder Following Somatic Motor Nerve Transfer
Source: Cells. 2025 Mar 11;14(6):406. doi: 10.3390/cells14060406 (PMC11941061; doi:10.3390/cells14060406)
Supplement: Supplementary file 1 [file cells-14-00406-s001.zip › cells-3410636-supplementary.pdf]

Figure S1. Diagram of Surgeries.

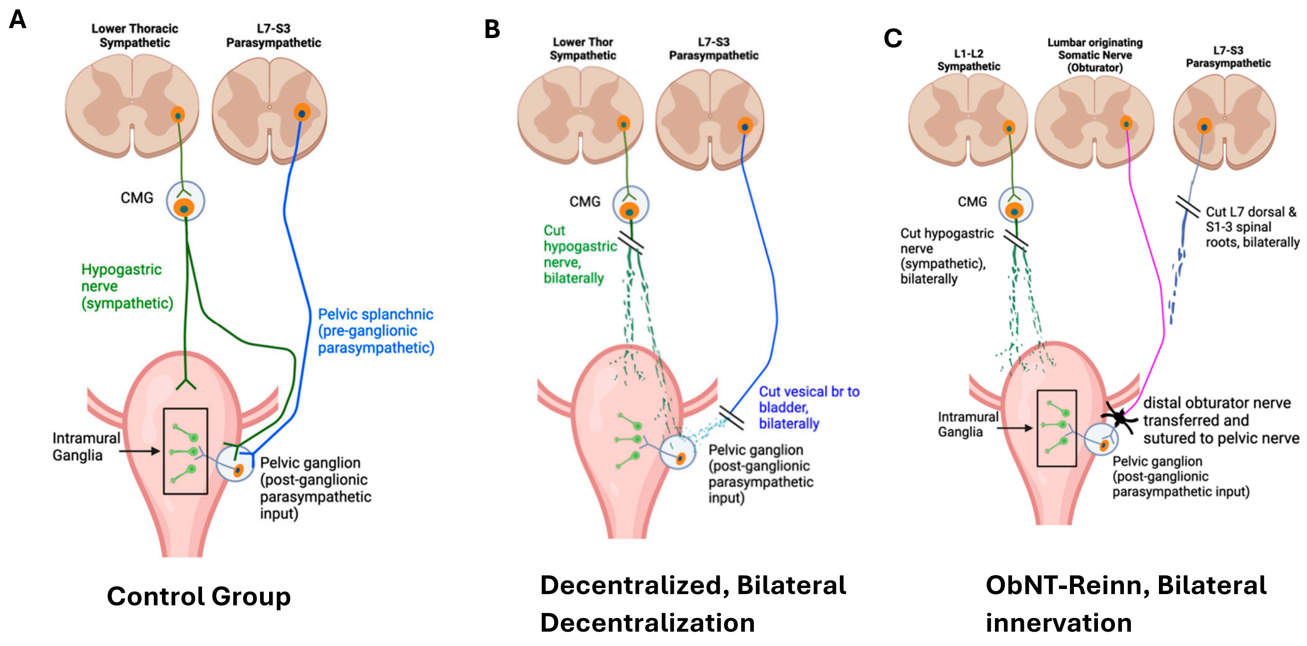

Figure S1. Anatomical Diagram of Groups: Control, Decentralized and Obturator Nerve Transfer and Reinnervation (ObNT-Reinn). Decentralization surgeries and nerve transfers were performed bilaterally. These figures were modified from a prior publication [51]. Images were created in https://BioRender.com

Figure S2

Enhanced BDNF and ROS in mucosa of lower motor neuron-lesioned dog bladder following somatic motor nerve transfer

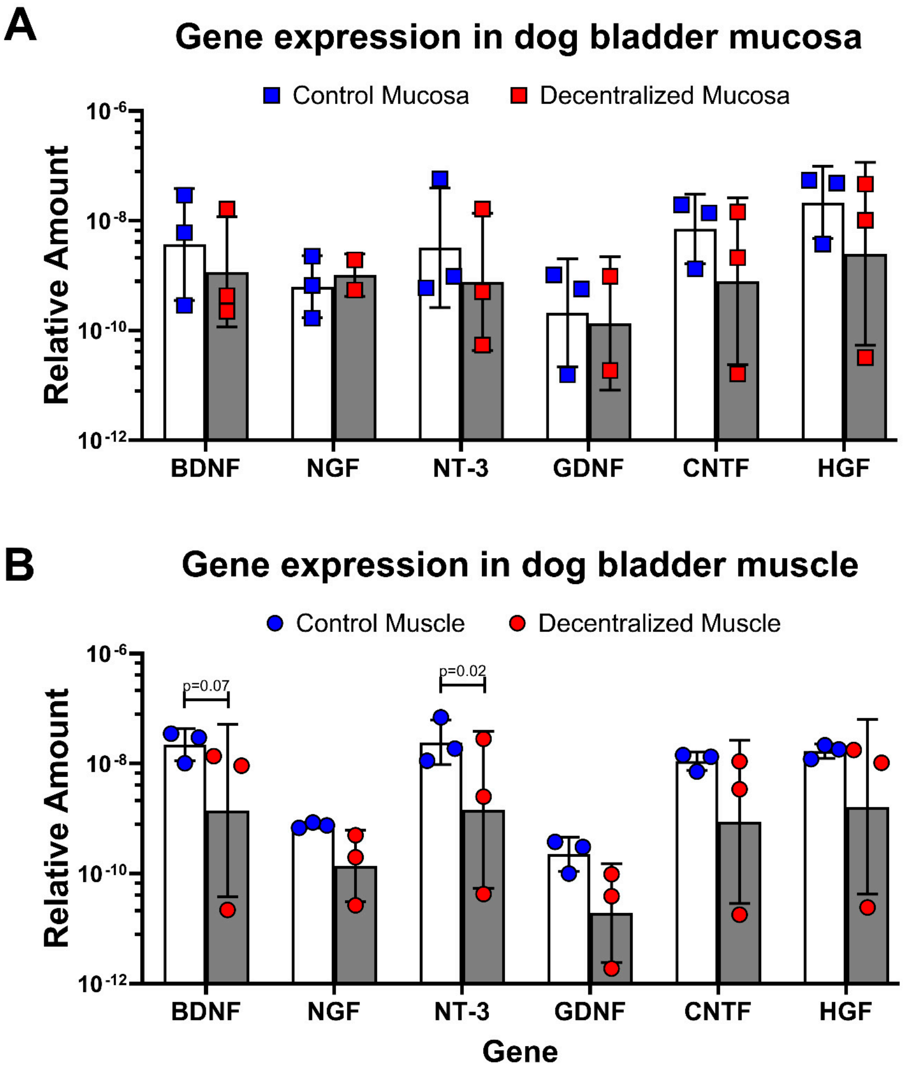

Figure S2. Expression of six neurotrophic factors-related genes in dog bladders. Relative amounts of RNA levels in the bladder tissues were obtained by transcription of DNA from RNA that was isolated from mucosa and muscle layers of the Control and Decentralized bladders. DNA was then quantified by real-time quantitative PCR using gene specific primers and analyzed, as described in the Materials and Methods section. Expression levels for each of the genes were calculated relative to the amount of RNA at the threshold cycle number. These levels were plotted on a logarithmic axis. We used the "absolute" values because there was large variation in the quantities of the b-actin reference gene used, suggesting that the levels of

its expression were affected by the surgical procedure. This choice allowed us to readily compare the levels of expression between each of the growth factors examined.

Supplemental Figure S3. Histological and Functional Data Outcomes

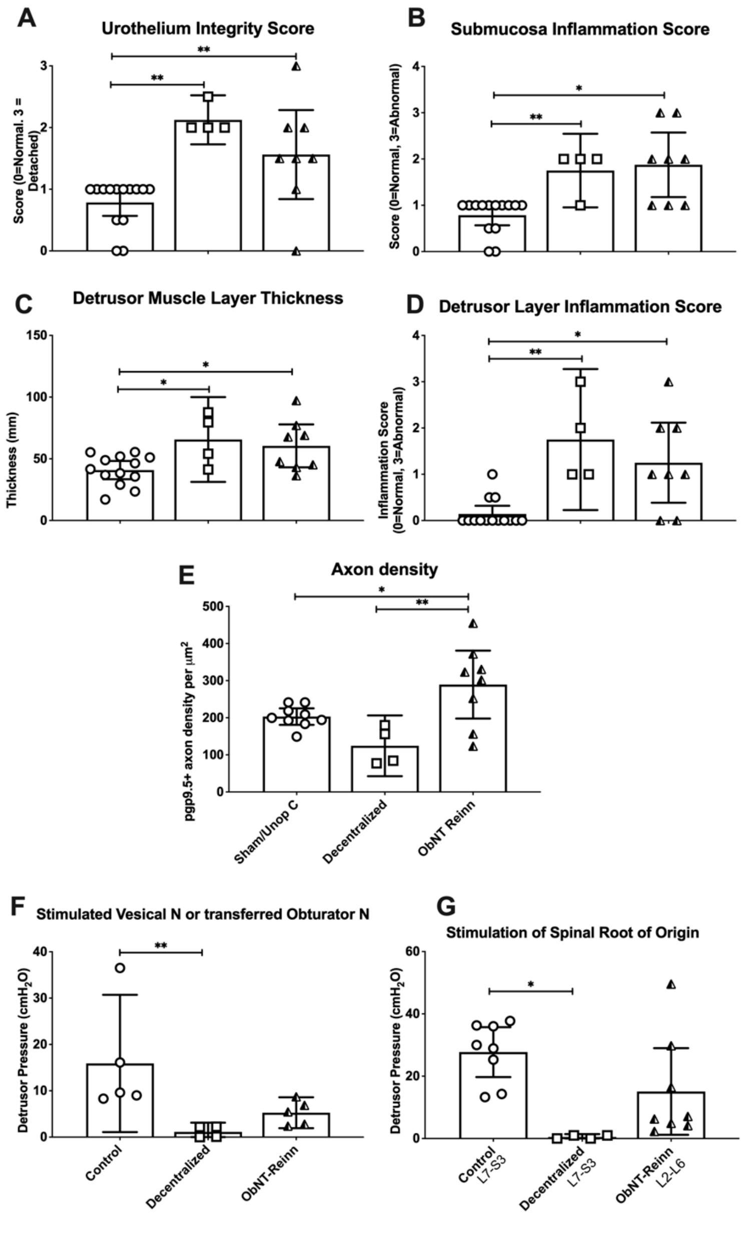

Supplemental Figure S3. Histological and functional outcomes from many of these same animals. These figures were modified from prior publications, which also include the methods used to gather this data [22,19]. Mean and 95% CI are shown. (A) Urothelium integrity score for which 0 = normal and 3 = detached. B) Submucosa inflammation score in which 0 = normal and 3 = abnormal (very inflamed). (C) Detrusor muscle layer thickness. (D) Detrusor muscle layer

inflammation score in which 0 = normal and 3 = abnormal (very inflamed). (E) Density of pgp9.5 (a pan neuronal marker) immunostained axons in the detrusor muscle layer. (F) Maximum detrusor pressure (MDP, cmH_2O) generated after pelvic nerve stimulation in Sham/Unop C, or after obturator nerve stimulation in Decentralized and ObNT Reinn. (G) Maximum detrusor pressure (MDP, cmH_2O) generated after stimulation of L2-L6 and L7-S3 spinal roots.
